# Reply to: “Inconsistent prediction capability of ImmuneCells.Sig across different RNA-seq datasets”

**DOI:** 10.1038/s41467-021-24304-4

**Published:** 2021-07-07

**Authors:** Donghai Xiong, Yian Wang, Ming You

**Affiliations:** Center for Cancer Prevention, Houston Methodist Cancer Center, Houston Methodist Research Institute, Houston, TX United States

**Keywords:** Cancer immunotherapy, Tumour biomarkers, Predictive markers

**Replying to** X. Xiao et al. *Nature Communication* 10.1038/s41467-021-24303-5 (2021).

In their commentary on our paper^1^, Xiao et al.^2^ make a point that the predictive performance of ImmuneCells.Sig is inconsistent. This is mainly due to the batch effect across different RNA-seq data sets (Fig. 1a). The relatively poor generalization of gene expression profiling (GEP) is common in predicting immunotherapy response. For example, Cui et al.^3^ analyzed ten well-established GEP signatures in the three data sets (VanAllen15, Liu19, Kim18). All ten signatures showed AUC (Area Under The Curve) values <0.66 and nine signatures had AUC < 0.6 in the Liu19 data set (Fig. 6D in Cui et al.^3^). The IMPRES signature^4^ performed poorly in all three data sets (AUC values are in the range of 0.5–0.63, Fig. 6D–F in Cui et al.^3^) and the AUC value of Messina signature is only about 0.2 in the Kim18 data set (Fig. 6F in Cui et al.^3^). Similarly, inconsistent and low AUC values of the established ICT (immune checkpoint therapy) response signatures were found in another study (Fig. 4g–i in Jiang et al.^5^). In addition, a study involving tumor specimens from 8135 patients and using the broad category of GEP developed from ten studies showed that the AUC value of this well-trained GEP is only 0.65^6^. It should be noted that some gene expression-based tests are successful in cancer diagnosis such as Oncotype DX for breast cancer. This is because that it is a tumor proliferation genes-based signature measured by a single reference laboratory^7^. Tumor proliferation genes’ expressions are highly correlated with cancer recurrence, so it is reasonable for Oncotype DX to predict the recurrence of breast cancer. Nevertheless, Oncotype DX could still perform poorly in predicting breast cancer outcome, with AUC values being 0.64 and 0.59 in two breast cancer data sets^8^.

**Fig. 1:**
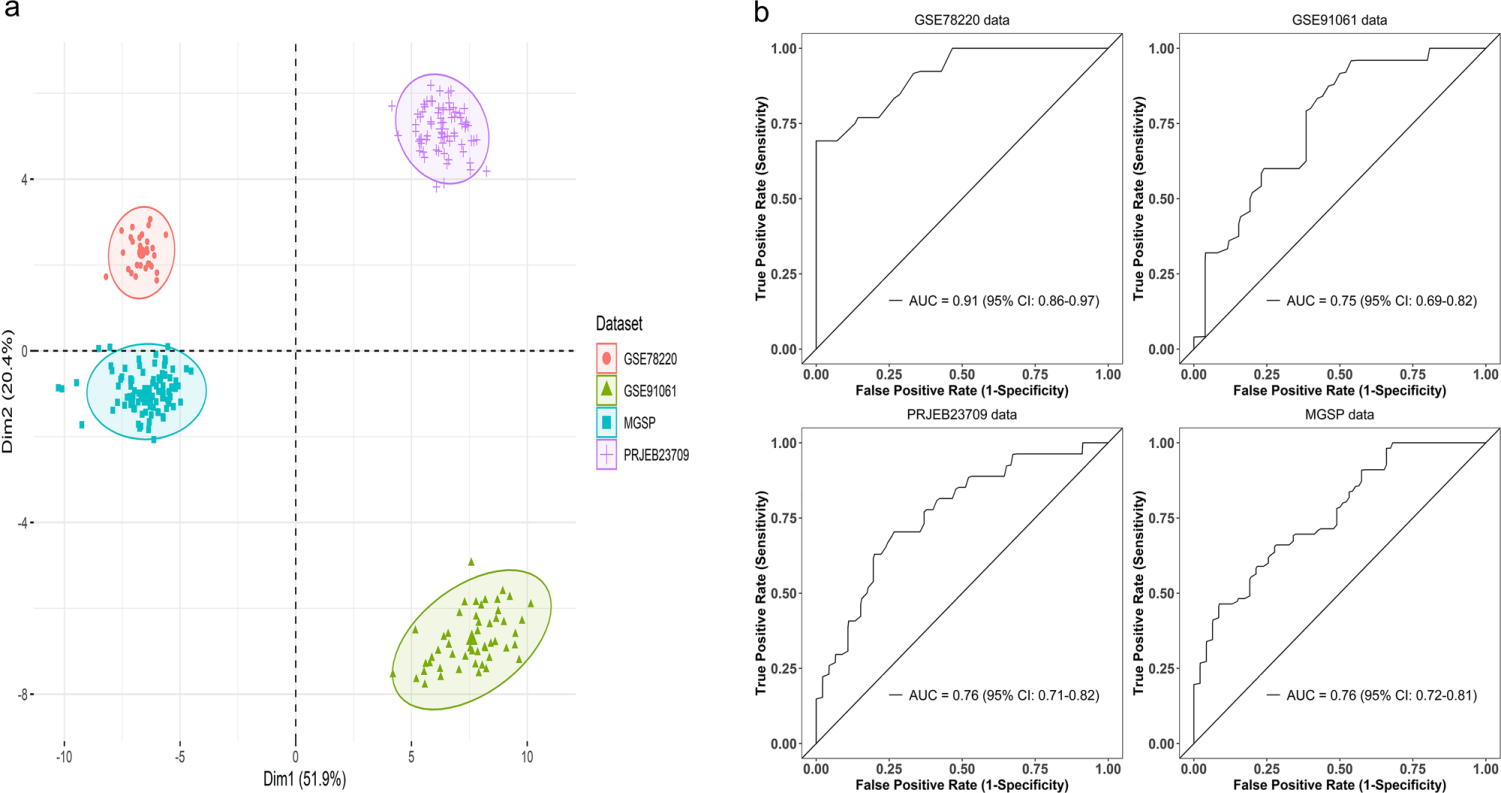
Divergence between different data sets and the fivefold cross-validation of ImmuneCells.Sig across the data sets. **a** First two principal components of all individuals from data sets GSE78220, GSE91061, PRJEB23709, and MGSP. **b** The fivefold cross-validation showed that the ImmuneCells.Sig still had good predictive values across the independent data sets. The plots of the results of the mean testing AUC values for each of the four data sets were shown, i.e., for GSE78220, GSE91061, PRJEB23709, and MGSP data sets.

In the four bulk RNA-seq data sets we used for validation, there existed the missing data problem. About 18% of the signature genes (19 of 108) had no expression data available in two or three bulk RNA-seq data sets. In addition, the patients received different treatment schemes that may result in the high heterogeneity of the biological samples. For example, for the GSE78220 data set^9^, patients received the anti-PD-1 therapy; for the PRJEB23709 data set^10^, the patients received either anti-PD-1 or combined anti-CTLA-4 and anti-PD-1 therapy; for the GSE91061 data set^11^, before the patients were treated with nivolumab (anti-PD-1), about half of them progressed on ipilimumab (Ipi) therapy (anti-CTLA-4) and the other half were Ipi-naive; for the MGSP study, some patients were exposed to the anti-CTLA-4 Ipi therapy while the others were Ipi-naive before they were treated with anti-PD-1 therapy. It could be difficult to accurately define ICT outcome, too. In some patients subjected to ICT, the durable responses may occur only after pseudoprogression that would be considered to be the disease progression phenotype^12^. Furthermore, the RNA samples were prepared differently across studies, with some being fresh samples and some being FFPE samples. Different bioinformatics approaches were used to process the sequencing data, too. All of these and other unknown factors contributed to the batch effects that hindered the generalization of the ImmuneCells.Sig signature we developed.

For the prediction, we were remiss to use the training AUC values for the comparison of the ICT response signatures. To correct this glitch, we have re-tested the predictive performance of ImmuneCells.Sig and the other 12 ICT signatures using the fivefold cross-validation^13^. To test a GEP signature in a data set, we split the data set into five parts of approximately equal size (called folds) and performed prediction for each part with a predictor trained on the remaining four parts. The mean testing AUC values from the fivefold cross-validation represent the generalization accuracy of a GEP signature. The results showed that ImmuneCells.Sig still had good predictive values (Fig. 1b). Comparing ImmuneCells.Sig to the other 12 ICT signatures suggested that the conclusion in our original paper remain valid, i.e., the ImmuneCells.Sig is better than the previously developed ICT signatures in predicting ICT outcomes (Fig. 2). For the data sets of GSE91061 and MGSP, ImmuneCells.Sig’s performance is obviously better than any of the other 12 ICT signatures (Fig. 2b, d). For the GSE78220 data set, ImmuneCells.Sig is one of the two best signatures (the other one is IPRES.Sig, Fig. 2a). For the PRJEB23709 data set, ImmuneCells.Sig is also one of the two best signatures (the other one is IMPRES.Sig, Fig. 2c). These results demonstrate that the ImmuneCells.Sig is an effective signature to predict ICT outcome.

**Fig. 2:**
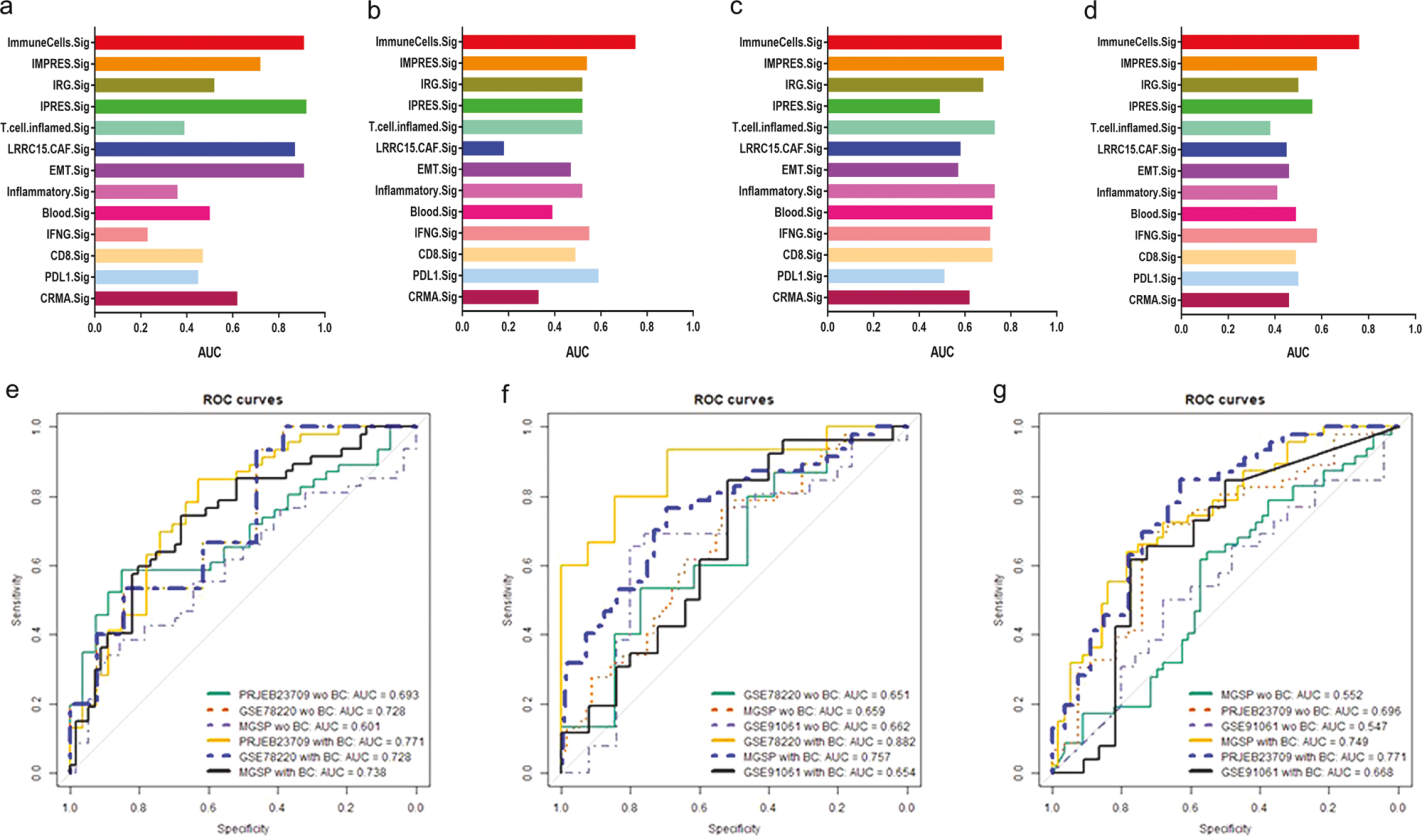
Comparison of the performance of ImmuneCells.Sig with other ICT (immune checkpoint therapy) response signatures and independent validation of ImmuneCells.Sig without or with batch effect correction. The multiple bar plots of the fivefold cross-validation calculated mean testing AUC (Area Under The Curve) values of the whole 13 ICT signatures are shown in **a** For the GSE78220 data set. **b** For the GSE91061 data set. **c** For the PRJEB23709 data set. **d** For the MGSP data set. **e** Testing the predictivity of the ImmuneCells.Sig trained in the GSE91061 data set in the other three independent data sets—PRJEB23709, GSE78220, and MGSP without or with batch effect correction. **f** Testing the predictivity of the ImmuneCells.Sig trained in the PRJEB23709 data set in the other three independent data sets—GSE78220, MGSP, and GSE91061 without or with batch effect correction. **g** Testing the predictivity of the ImmuneCells.Sig trained in the GSE78220 data set in the other three independent data sets—MGSP, PRJEB23709, and GSE91061 without or with batch effect correction.

In addition, we reanalyzed the data using the regularized logistic regression method according to the previous studies^14,15^. The AUC value within 0.7–0.8 and 0.8–0.9 is considered acceptable and excellent, respectively^16^. The new results showed that the ImmuneCells.Sig was validated in the independent testing data sets for prediction. If we corrected the batch effect using the removeBatchEffect function in the limma package v3.44.3 implemented in the R software package v3.6.3, the AUC values were further improved. For example, without batch effect correction, the ImmuneCells.Sig trained in the GSE91061 data set was validated in the independent test data sets—PRJEB23709 and GSE78220, which achieved the AUC values of 0.693 and 0.728, respectively (Fig. 2e). It was not validated in the MGSP data set without batch effect correction (AUC = 0.6, Fig. 2e). After batch effect correction, the ImmuneCells.Sig was validated in these test data sets. The AUC values were 0.771, 0.728, and 0.738 for the batch effect corrected PRJEB23709, GSE78220, and MGSP data sets, respectively (Fig. 2e). The PCA plots of batch effect correction were given in Supplementary Fig. 1. Except for the GSE78220 data set whose AUC value remained 0.728, ImmuneCells.Sig performance was improved in the other two test data sets after batch effect correction. Specifically, in the PRJEB23709 data set, the acceptable AUC value was improved to a higher value (from 0.693 to 0.771); in the MGSP data set, the AUC value in the batch effect corrected data increased to the acceptable level (from 0.6 to 0.738).

Similar improvement was also seen for the ImmuneCells.Sig trained in the PRJEB23709 data set. Without batch effect correction, ImmuneCells.Sig trained in the PRJEB23709 data set achieved AUC values of 0.651, 0.659, 0.662 in the three independent test data sets—GSE78220, MGSP, and GSE91061. With batch effect correction, the AUC values reached 0.882, 0.757, 0.654, respectively, for these data sets. Except for GSE91061, ImmuneCells.Sig was validated in the corrected GSE78220 and MGSP data sets, at the excellent (0.882) and acceptable (0.757) levels, respectively (Fig. 2f). A similar effect was also observed for ImmuneCells.Sig trained in the GSE78220 data set (Fig. 2g). The AUC values were 0.552, 0.696, and 0.547 in the initial test data sets—MGSP, PRJEB23709, and GSE91061. They increased to the acceptable validation levels of 0.749 and 0.771 for the batch effected corrected MGSP and PRJEB23709 data sets and reached 0.668 for the corrected GSE91061 data set that was close to the acceptable validation (Fig. 2g). Therefore, batch effect correction could improve the prediction performance of ImmuneCells.Sig to the acceptable level.

It is a known issue that batch effects of heterogeneous gene expression data sets greatly impair the generalization of predictive models trained in one data set to other data sets^17,18^. The use of fivefold cross-validation, batch effect correction, and regularized logistic regression defended the prognostic values of ImmuneCells.Sig in predicting ICT response proposed in our original study^1^.

## Methods

### Principal component analysis and batch effect correction

The four bulk RNA-seq data sets (GSE78220, GSE91061, PRJEB2370922, and MGSP), our self-developed gene expression signature—ImmuneCells.Sig and the twelve other published gene expression signatures for comparison in our original publication^1^ were also used in this study. Principal Component Analysis (PCA) was conducted using the factoextra R package v1.0.7. To correct the batch effect, we utilized the removeBatchEffect function in the limma package v3.44.3 implemented in the R software package v3.6.3.

### Data analysis

For validation study of the accuracy of the gene expression signature—ImmuneCells.Sig in predicting ICT outcome, we reanalyzed the data using the regularized logistic regression methods according to the previous studies^14,15^. The custom codes for applying the regularized logistic regression methods to our own data were developed based on the modification of the original codes kindly provided by Dr. Zhi-Ping Liu from a previous study^14^.

### Reporting summary

Further information on research design is available in the Nature Research Reporting Summary linked to this article.

## Supplementary information

Supplementary Information

Reporting Summary

## Data Availability

GES accession codes for the first two data sets used in this reply study are GSE78220 and GSE91061. The third data set PRJEB2370922 was retrieved from the website link—https://www.ebi.ac.uk/ena/data/view/PRJEB23709. The fourth data set—MGSP was available in dbGaP under accession number phs000452.v3.p1. The data files generated during the processing of the above raw data sets are freely available in our GitHub repository https://github.com/donghaixiong/Immune_cells_analysis.

## Code availability

The code for our new computation related to the figures has been uploaded in the GitHub repository (https://github.com/donghaixiong/ReplyToMattersArising). The corresponding DOI is as follows 10.5281/zenodo.4717985.
