# Supplementary material for: Reply to: “Inconsistent prediction capability of ImmuneCells.Sig across different RNA-seq datasets”
Source: Nat Commun. 2021 Jul 7;12:4168. doi: 10.1038/s41467-021-24304-4 (PMC8263738; doi:10.1038/s41467-021-24304-4)
Supplement: Supplementary file 1 — Supplementary Information [file 41467_2021_24304_MOESM1_ESM.pdf]

Supplementary Information

Reply to 'Inconsistent prediction capability of ImmuneCells.Sig across different RNA-seq datasets'

Xiong et al.

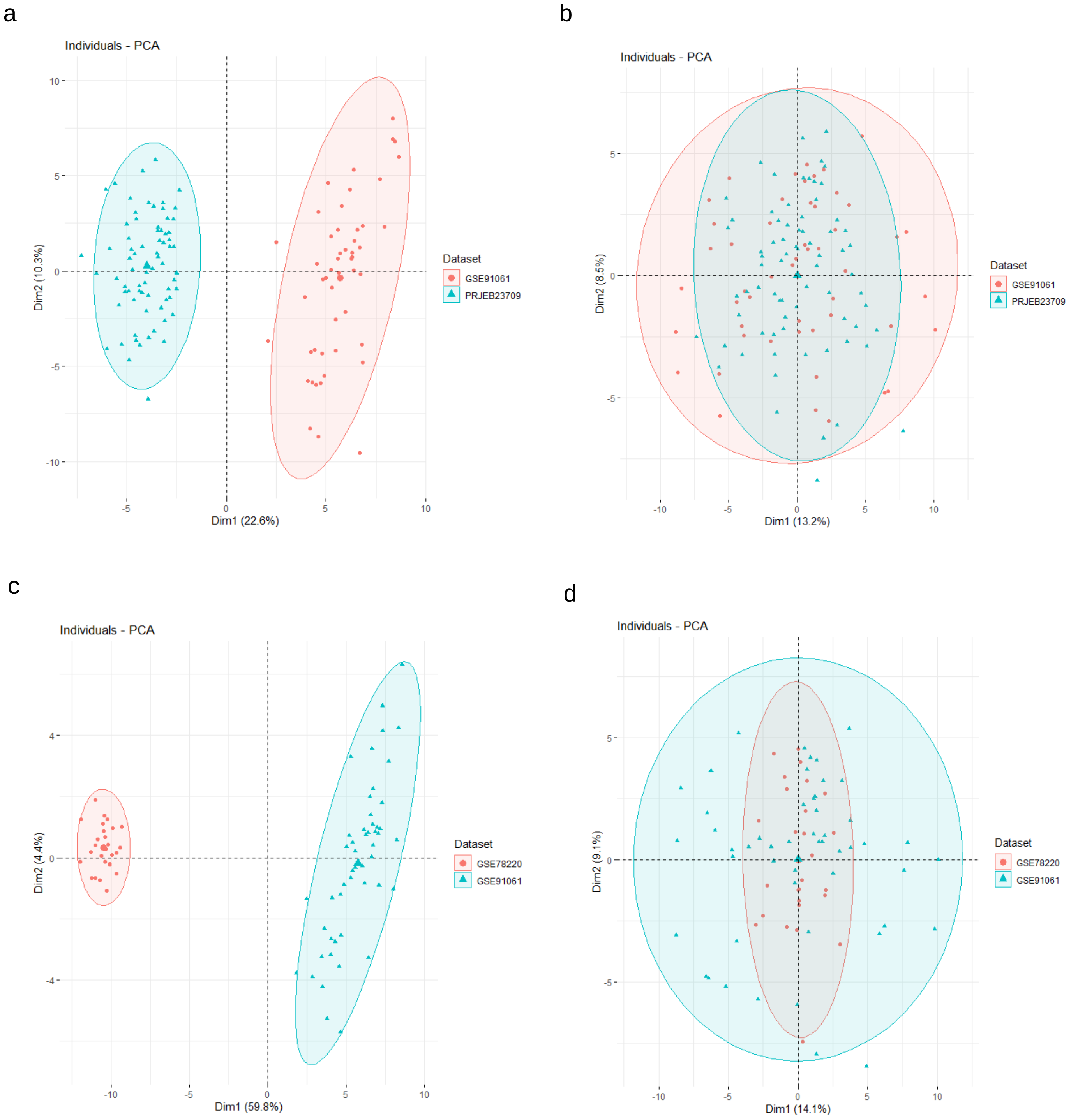
a
Individuals - PCA
Dim2 (10.3%)
Dim1 (22.6%)
Dataset
GSE91061
PRJEB23709
b
Individuals - PCA
Dim2 (8.5%)
Dim1 (13.2%)
Dataset
GSE91061
PRJEB23709
c
Individuals - PCA
Dim2 (4.4%)
Dim1 (59.8%)
Dataset
GSE78220
GSE91061
d
Individuals - PCA
Dim2 (9.1%)
Dim1 (14.1%)
Dataset
GSE78220
GSE91061

e

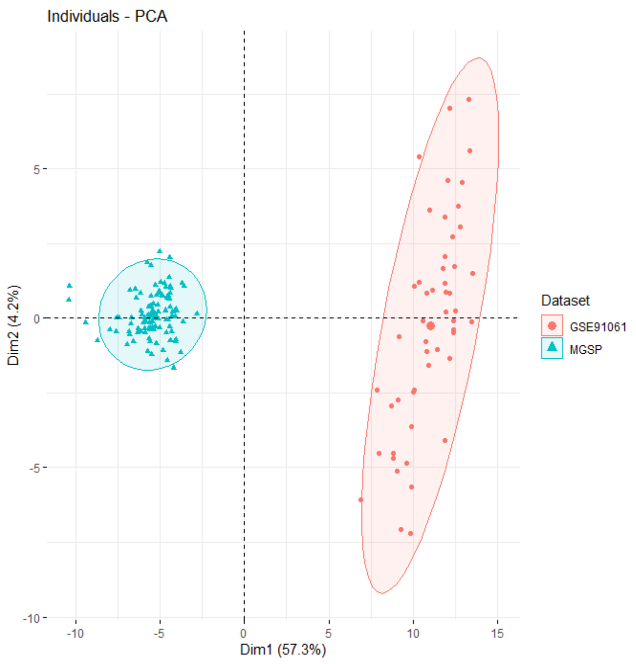

f

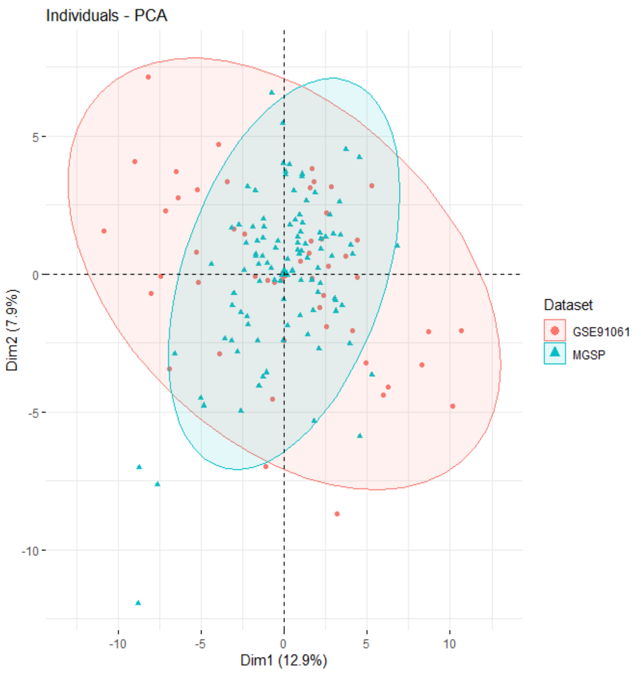

Supplementary Figure 1. PCA plots of batch effect correction for pairwise datasets with GSE91061 being the training dataset for ImmuneCells.Sig and the other being the testing dataset. (a) PCA plot of original GSE91061 and PRJEB23709 datasets; (b) PCA plot of batch effect corrected GSE91061 and PRJEB23709 datasets; (c) PCA plot of original GSE91061 and GSE78220 datasets; (d) PCA plot of batch effect corrected GSE91061 and GSE78220 datasets; (e) PCA plot of original GSE91061 and MGSP datasets; (f) PCA plot of batch effect corrected GSE91061 and MGSP datasets.
