# Supplementary material for: Reply to: “Inconsistent prediction capability of ImmuneCells.Sig across different RNA-seq datasets”
Source: Nat Commun. 2021 Jul 7;12:4168. doi: 10.1038/s41467-021-24304-4 (PMC8263738; doi:10.1038/s41467-021-24304-4)
Supplement: Supplementary file 2 — Reporting Summary [file 41467_2021_24304_MOESM2_ESM.pdf]

nature research

Corresponding author(s): Ming You

Last updated by author(s): Apr 22, 2021

Reporting Summary

Nature Research wishes to improve the reproducibility of the work that we publish. This form provides structure for consistency and transparency in reporting. For further information on Nature Research policies, see our Editorial Policies and the Editorial Policy Checklist.

Statistics

For all statistical analyses, confirm that the following items are present in the figure legend, table legend, main text, or Methods section.

n/a	Confirmed	
☐	☒	The exact sample size (n) for each experimental group/condition, given as a discrete number and unit of measurement
☒	☐	A statement on whether measurements were taken from distinct samples or whether the same sample was measured repeatedly
☒	☐	The statistical test(s) used AND whether they are one- or two-sided<br>*Only common tests should be described solely by name; describe more complex techniques in the Methods section.*
☒	☐	A description of all covariates tested
☒	☐	A description of any assumptions or corrections, such as tests of normality and adjustment for multiple comparisons
☐	☒	A full description of the statistical parameters including central tendency (e.g. means) or other basic estimates (e.g. regression coefficient) AND variation (e.g. standard deviation) or associated estimates of uncertainty (e.g. confidence intervals)
☐	☒	For null hypothesis testing, the test statistic (e.g. F, t, r) with confidence intervals, effect sizes, degrees of freedom and P value noted<br>*Give P values as exact values whenever suitable.*
☒	☐	For Bayesian analysis, information on the choice of priors and Markov chain Monte Carlo settings
☒	☐	For hierarchical and complex designs, identification of the appropriate level for tests and full reporting of outcomes
☒	☐	Estimates of effect sizes (e.g. Cohen's d, Pearson's r), indicating how they were calculated

Our web collection on statistics for biologists contains articles on many of the points above.

Software and code

Policy information about availability of computer code

Data collection	No software was used for data collection
Data analysis	R software, version 3.6.3 (R Project for Statistical Computing); The limma package v3.44.3 ; Principal Component Analysis (PCA) was conducted using the factoextra R package v1.0.7. The R codes for our new computation related to the figures 1 and 2 have been uploaded in the GitHub repository web link - https://github.com/donghaixiong/ReplyToMattersArising

For manuscripts utilizing custom algorithms or software that are central to the research but not yet described in published literature, software must be made available to editors and reviewers. We strongly encourage code deposition in a community repository (e.g. GitHub). See the Nature Research guidelines for submitting code & software for further information.

Data

Policy information about availability of data

All manuscripts must include a data availability statement. This statement should provide the following information, where applicable:

- Accession codes, unique identifiers, or web links for publicly available datasets
- A list of figures that have associated raw data
- A description of any restrictions on data availability

GES accession codes for the first two datasets used in this reply study are GSE78220 (https://www.ncbi.nlm.nih.gov/geo/query/acc.cgi?acc=GSE78220) and GSE91061 (https://www.ncbi.nlm.nih.gov/geo/query/acc.cgi?acc=GSE91061). The third dataset PRJEB2370922 was retrieved from the website link - https://www.ebi.ac.uk/ena/data/view/PRJEB23709. The fourth dataset - MGSP was available in dbGaP under accession number phs000452.v3.p1. The corresponding web link is: https://www.ncbi.nlm.nih.gov/projects/gap/cgi-bin/study.cgi?study_id=phs000452.v3.p1. All the other data used in this study are freely available in our GitHub repository: https://github.com/donghaixiong/Immune_cells_analysis.

Field-specific reporting

Please select the one below that is the best fit for your research. If you are not sure, read the appropriate sections before making your selection.

- [x] Life sciences
- [ ] Behavioural & social sciences
- [ ] Ecological, evolutionary & environmental sciences

For a reference copy of the document with all sections, see nature.com/documents/nr-reporting-summary-flat.pdf

Life sciences study design

All studies must disclose on these points even when the disclosure is negative.

Sample size	This reply study is based on the analyses of the publicly available datasets. Four bulk RNA-seq datasets from 255 samples (28+51+73+103) of cancer patients subjected to immune checkpoint therapy were analyzed.
Data exclusions	This study is based on the analyses of the publicly available datasets. No data in those previous studies were excluded from the analyses.
Replication	No replication possible as study used clinical trial samples.
Randomization	Our reply study is based on the four immune checkpoint therapy studies previously published. In those studies, patients were recruited to the associated clinical trial of immune checkpoint therapy and no randomization was used for patient samples, since all patients received immune checkpoint therapy, and no control (no therapy) samples were analyzed in the studies.
Blinding	No blinding was used for patient identity, since all patients received immune checkpoint therapy, and no control (no therapy) samples were analyzed in this study.

Reporting for specific materials, systems and methods

We require information from authors about some types of materials, experimental systems and methods used in many studies. Here, indicate whether each material, system or method listed is relevant to your study. If you are not sure if a list item applies to your research, read the appropriate section before selecting a response.

Materials & experimental systems

n/a	Involved in the study	
x		Antibodies
x		Eukaryotic cell lines
x		Palaeontology and archaeology
x		Animals and other organisms
x		Human research participants
x		Clinical data
x		Dual use research of concern

Methods

n/a	Involved in the study	
x		ChIP-seq
x		Flow cytometry
x		MRI-based neuroimaging
